# Creating falseness—How to establish statistical evidence of the untrue

**DOI:** 10.1111/jep.12823

**Published:** 2017-09-27

**Authors:** Per Lytsy

**Affiliations:** ^1^ Department of Public Health and Caring Sciences Uppsala University Uppsala Sweden

**Keywords:** epidemiology, randomized controlled trials, statistics

## Abstract

Null hypothesis significance testing is the typical statistical approach in search of the truthfulness of hypotheses. This method does not formally consider the prior credence in the hypothesis, which affects the chances of reaching correct conclusions. When scientifically implausible or empirically weakly supported hypotheses are tested, there is an increased risk that a positive finding in a test in fact is false positive. This article argues that when scientifically weakly supported hypotheses are tested repeatedly—such as when studying the clinical effects of homeopathy—the accumulation of false positive study findings will risk providing false evidence also in systematic reviews and meta‐analyses. False positive findings are detrimental to science and society, as once published, they accumulate persistent untrue evidence, which risks giving rise to nonpurposive research programmes, policy changes, and promotion of ineffective treatments. The problems with false positive findings are discussed, and advice is given on how to minimize the problem. The standard of evidence of a hypothesis should depend not only on the results of statistical analyses but also on its a priori support. Positive findings from studies investigating hypotheses with poor theoretical and empirical foundations should be viewed as tentative until the results are replicated and/or the hypothesis gains more empirical evidence supporting it as likely to be true.

## 1 BACKGROUND

The ultimate goal of science is to build knowledge about the world, where knowledge intrinsically is understood to be true. In the world of quantitative analyses, the frequentist null hypothesis significance test (NHST) has become the operative standard of statistical inference in search of that truth. Despite vivid attacks on the legitimacy of this method,^1^, ^2^, ^3^, ^4^, ^5^ it is certainly a well‐established approach in the mission of trying to separate true hypotheses from false. Arguably, it may also be used to create evidence of the untrue. Just follow this step‐by‐step guide on how to establish statistically significant evidence of a noneffective treatment.

### 1.1 The theory

First, decide upon a theory that is simply not likely to be true (not true by the definition in our example). Choose wisely and base it preferably on a story that bears an aura of ancient medical wisdom. Why not pick the idea of Hippocrates, the founding father of medicine, who believed in so‐called humoral medicine, the notion that health is determined by the balance of the 4 body fluids: black and yellow bile, red blood, and blue phlegm. This fine theory bears some apparent validity, as most medically educated persons would agree that a distortion of the proportions of these fluids, at some point, will be associated with the onset of ill health.

### 1.2 The treatment

When choosing a treatment, it is advisable to avoid active ingredients. There are several reasons for this. Few molecular compounds tend to have desirable biological health effects, at increasing dosages most are yet likely to cause several unwanted effects. Drugs with no biological effects are much more favourable in that sense.

As the treatment lacks any true effect, it is, however, in need of some symbolic loading. According to knowledge within the humoral‐pathological paradigm, the body fluids possess different natures: They are either hot or cold and could also be dry or moist. This naturally implies that pills produced under such different environmental circumstances are likely to possess therapeutic properties of interest. Subsequently, with an apparent logic, we now have a colourful therapeutic arsenal suitable for humoral amendment.

### 1.3 The clinical encounter

People vary in all kinds of ways, which naturally also goes for their body fluid imbalance and its need of adjustment. Consequently, all therapeutic strategies must be individualized according to the standards of humoral medicine. One patient may require 12.5 units of a cold blue drug in combination with just mere 2.5 units of a dry black drug (to be re‐evaluated and dose‐adjusted at the next clinical visit). Others are in need of their specific therapeutic combinations, according to the discretion of each humoral clinician. The individualized approach is essential to humoral medicine, as it addresses the holistic and true aetiology of human ill health and not merely aims at temporary symptomatic relief.

### 1.4 The research

Yes, do encourage research! The individualized treatment approach naturally adds complexity to the evaluation practice, but the truth deserves to be multifaceted if that is its nature. This also contrasts humoral medicine with the allopathic paradigm and its obsessions with rigid diagnoses, specific treatment protocols, and fixed doses. Patient satisfaction is, however, never wrong to evaluate, and incidental and anecdotal evidence of miraculous health improvements will accumulate with time.

Do not hesitate to encourage research according to rigorous standards, investigating the effect of humoral medicine in randomized controlled trials. When doing so, rely on the inherent powers of the NHST method, which is not anchored by prejudices related to your hypothesis. In fact, the commonly used significance level of 0.05 will over time surely produce the anticipated results in about 5% of the one‐sided tests performed,^6^ in these studies clearly demonstrating the superiority of humoral medicine over a placebo.

Furthermore, have trust in the creativity within the research community. When one test disappointingly turns out not to reach statistical significance, others indisputably will. It is just a matter of retesting your hypothesis in a slightly different way, in a somewhat different population until that truth is revealed.^7^ Some researchers may even sensibly value the integrity of humoral medicine higher than the tedious procedures of blinded study protocols, just adding a touch of bias or even data modification^8^ for the sake of a good cause.

Be prepared for an acrimonious publishing process. Hard‐core journals are expected to reject most findings using arguments as “implausible” and even “ridiculous.”

This is a good thing, however, as the vast body of negative findings will be regarded as expected and trivial, thus never reaching any indexed research database. Positive findings on the other hand, refreshing and valiant in their spirit, bring hope of a new understanding of the world, even when published in journals with lesser impact.

### 1.5 The meta‐analysis

With time, the combination of multiple analyses by creative researchers 6, 7 false positive findings through type 1 errors^9^, ^10^ and the steering forces of publication bias^11^, ^12^ will work in your favour. Eventually, there will be a comprehensive yet inconsistent body of evidence seemingly supporting the effect of humoral medicine, at least to some extent. At this point, it is time to gather the evidence in a systematic review and meta‐analysis. Note that this is important work, not to be performed and supervised by anyone else than an internationally acknowledged professor with indisputable scientific merits. Further, the study protocol must be predefined and follow the highest of standards. Do not wait too long to initiate this process as there should not be enough studies to enable separate meta‐analyses on different medical conditions. Thus, all randomized controlled trials investigating humoral treatment efficacy will be used to statistically answer one question: Does it really work?

Even though the number of negative findings in such a systematic search may rule out the number of positive ones, remain faithful to the statistical procedure, as even trivial differences will turn out to be statistically significant when the sample size is large enough.^13^ The inclusion of studies with vastly different outcomes, measured in different ways at different points in time for entirely different medical conditions, perfectly serves your objective. When the meta‐analysis arrives at a statistically significant odds ratio of, let us say, 1.82 (95% CI, 1.21‐2.52, *P* = .0076), no sensible person alive will be able to interpret what that means, nor if it has any clinical significance. The result does not apply to any specific disease, problem, treatment regimen, or hypothesis; rather, it will be interpreted as support of humoral medicine as a whole. This is a well‐adopted analytic strategy in meta‐analyses assessing the effect of nonallopathic regimens.^14^, ^15^, ^16^

In the absence of methodological flaws in such a meta‐analysis, reviewers, editors, and the whole world will just have to accept the facts. And this is when humoral medicine, despite its shortcomings of being in fact ineffective, will enter the fame as an, beyond reasonable statistical doubts, evidence‐based medical treatment.

## 2 DISCUSSION

The goal of science is to find out something about the world that is true, not untrue. Unreflected use of classical statistical hypothesis testing might, however, increase the risk of the latter. Ever since the NHST was formed as a hybrid of Fisher and Neyman/Pearson different frequentist approaches,^1^ there has been an ongoing debate about the method's shortcomings. The objections have included not only the philosophical and logical validity of the method^17^, ^18^ but also the widespread misunderstandings of the interpretation of the *P* value,^2^, ^4^ including the unfortunate circumstance that it is not a valid indicator of what we want to know: whether our hypothesis is true or false.

Yet another fundamental, but in medical studies rarely acknowledged, weakness of the NHST method is that it disregards biological understanding and previous research results of the hypothesis it is testing.^18^ While this to some may sound like a good thing—a test that is not prejudicious about the analysed data—it actually has profound consequences on the chances of making correct inferences about how the world works. What we want to find out is whether the hypothesis is likely to be true or not, which in the light of a well performed study depends on 3 factors^19^: (1) the significance level used; (2) the power of the study; and (3) the prior probability of the tested hypothesis. The NHST handles the first two of these issues: by convention a significance level of 5% and a power of 80% are often thought of as balanced and accepted risks of making types 1 and 2 errors, that is, producing false positive or false negative results, respectively. The NHST, however, does not formally consider the third point: the prior probability that the hypothesis is true. This is foolish, even ignorant, as hypotheses are not created equal.^18^

Suppose that you are asked to investigate 2 new drugs, A and B, and their preventive effect on a disease, D. You have no prior information about the drugs, but apparently, the adverse effects are believed to be mild. You design 2 large, equally sized, state‐of‐the‐art, randomized controlled trials where the drugs separately are compared to a placebo. When the studies are completed, the results happen to be identical for A and B; in both studies, the drugs have been shown to prevent the outcome with an odds ratio of 1.76 (95% CI, 1.21‐2.31, *P* = .0036).

Time for statistical inference. Do these figures provide evidence that the treatments work? Do they support the test hypotheses to be true? Do the results imply that the underlying hypotheses of A and B are equally likely to be true?

Leaning on the notion that evidence from NHSTs provides some sort of fact that is likely to be true, it is tempting to respond “yes” to these questions.

Now, consider that you get some additional information once the trial results have already been analysed and presented. It turns out that treatment A is a well‐described pharmacological substance with a biologically known mode of action at a receptor level. Pharmacodynamics support that drug A has a dose‐response relationship to some physiological system relevant to disease D, and pharmacokinetics support that drug A has a bioavailability seemingly appropriate compared to other similar and effective drugs. Furthermore, there are a dozen phases I and II, and even one smaller phase III study, all in support that drug A has an appropriate effect.

Drug B is a homeopathic remedy. A mother tincture, once containing a herb believed to be used for health reasons by Inca Indians, has been ultradiluted to the point where no known scientific method can distinguish it from a placebo. There are no studies of the pharmacological properties of drug B, and no studies support the existence of a biological effect. In fact, the idea that an ultradiluted remedy could have an effect, other than as a placebo, has been deemed scientifically implausible.^20^

Would you, in the light of the new background information, be willing to alter the views that drugs A and B are equally likely to truly work? If you respond *yes* to that question, you are wiser than the NHST method, because you value the study findings in the light of the scientific context, including the previous evidence and the treatments' biological plausibility. That is undeniably a sensible way to reason. A hypothesis may be either true or false, but we will never—regardless of the amount of evidence gathered—be absolutely sure, an inherent limitation of statistical inductive logic. We will, however, be able to gather more and more evidence guiding us whether our hypothesis is true or not, and this evidence forms our beliefs. The higher we deem a hypothesis likely to be correct—on empirical and plausible grounds—the higher the probability that a positive finding from an NHST is actually true.^21^ And vice versa, when a seemingly implausible or empirically weakly supported hypothesis is scrutinized under the NHST, there is a substantial risk that a positive finding in fact is false positive, ie, false evidence.^19^, ^21^

**Figure 1:**
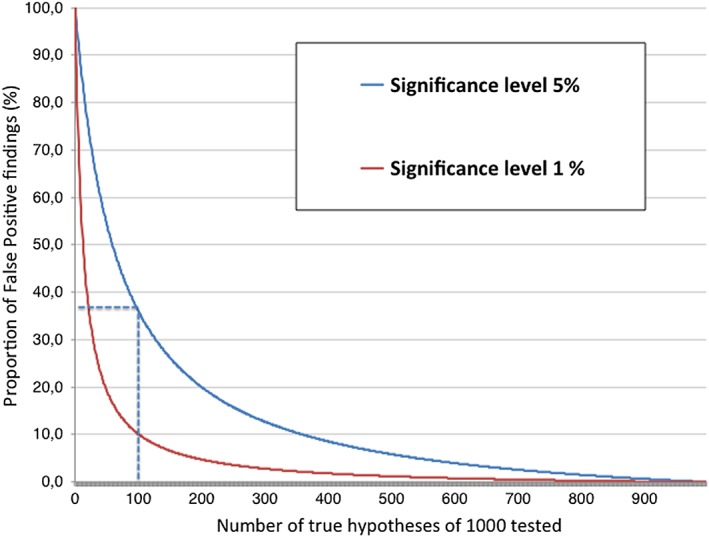
Proportion of false positive findings (y‐axis) to proportion of true hypotheses tested (x‐axis) using 80% power and significant levels (α) of 0.05 and 0.01, respectively

While the NHST procedure does not formally consider the prior probability of the tested hypothesis, the risk of reaching a false positive conclusion may be modelled when testing many hypotheses, given a known proportion of true and false hypotheses tested. For example, if you use conventional levels of significance and power (5% and 80%, respectively) and test 1000 hypotheses of which 100 are actually true, you are expected to arrive at about 125 positive findings (80 true positives and 45 false positives). Thus, a randomly chosen positive test finding has about a 36% risk of being untrue. As illustrated in Figure 1, the risk of creating false evidence increases sharply with a lower prior likeliness of a tested hypothesis.

The fact that the prior likeliness of a hypothesis affects the ability of making a correct inference is analogous with the use of clinical diagnostic tests.^22^ The likeliness that a positive test finding in a diagnostic procedure identifies a truly sick person depends not only on the test's accuracy (sensitivity and specificity) but also on the prevalence (the prior likeliness) of the disease in the tested population that the person belongs to.^22^ For this reason, mass screening is typically not recommended in low‐prevalence populations, as it would incorrectly classify many healthy individuals as sick, ie, providing false positives.^23^

This paper uses homeopathy as an example of a treatment with a low prior likeliness to work. Homeopathy is illustrative, as there is no reliable evidence of a mechanism of action, thus suggesting that published positive treatment effect findings are likely to be false positives. How valid is the same argument in regard to more mainstream medical hypotheses regularly tested using the NHST? It is difficult to give a clear answer since formal assessments of the prior probably of the test hypothesis lies outside the frequentist paradigm. Even if there is poor understanding of how a treatment might work (such as the case with homeopathy), the NHST may still be appropriate to use when other scientific evidence support the existence of a true effect. For example, the mechanism of some general anaesthetics and psychoactive drugs are yet not fully understood in detail, but preclinical and clinical data may strongly support the existence of true effects, for example, by existing dose‐response relationships.

The risk of reaching false conclusions may vary within different fields of research. It has been argued that “hotter” research fields (where many research teams compete to be first of novel findings) and that research fields driven by strong financial interests may increase the pursue of “positive results,”^19^ which later are refuted by other research teams unable to replicate the results.^24^ Other research fields regularly testing low prior likeliness hypotheses include alternative and integrative treatment approaches, which many times are based on treatment ideas not compatible with basic scientific and medical paradigms.

The recent and ongoing debate about a replication crisis is about that many significant research findings are difficult to replicate, possibly partly because of initial false positive findings. This seems to be true in test simulations,^25^ in psychological research^26^ as well as in laboratory economic research.^27^ No large‐scale replication study has yet been performed on medical studies, but reports from the industry suggest that many initial new drug target claims are rarely reproducible.^28^

## 3 HOW TO REDUCE FALSE POSITIVE FINDINGS

False positive research findings are perhaps the most unfortunate error a scientist can make, as once published, they are particularly persistent.^7^ Even if replication attempts fail and the results get published, such negative findings will never be fully convincing, as a failure to reject a null hypothesis constitutes no evidence that the null is true. The cumulative evidence will, thus, be ambiguous, and it will waste resources if giving rise to pointless investments and research programmes. False positive findings may further harm society, organizations, and ultimately individuals if they spawn nonpurposive policy changes and ineffective treatments.^7^

When choosing a statistical approach to investigate a hypothesis, it is wise to choose one prone to result in a correct inference, which includes a minimization of the risk of reaching a false positive conclusion. To do this, the prior likeliness of the hypothesis must be considered. If a hypothesis is deemed implausible or if it does not conform to established science or if it just has weak empirical support, consider one of the following approaches to reduce the risk of producing evidence of the untrue:

- Use the Bayesian approach of statistical inference. This has been suggested repeatedly,^18^, ^29^, ^30^ also in combination with classical statistical hypothesis testing.^31^, ^32^ Among the virtues of the Bayesian method is that it gives an estimate of what you really want to know: the likeliness that your hypothesis is true. This method balances the test study findings with the previous knowledge in the area. Yes, it may be difficult to agree on the quantification of the prior belief in the hypothesis. Yet to avoid doing so (which is the case within the procedure of NHST) is treating existing knowledge of a tested hypothesis as noninformative, which is an unfortunate kind of self‐imposed systematic scientific ignorance.
- If an NHST is performed on a weakly supported hypothesis, consider the use of stricter significance levels, as this will reduce the risk of producing false positive findings. The choice of significant level should, apart from the prior evidence, take several factors in account, including the number of tests performed, the size of the study population, and on the consequences on any the conclusions of the test. A significance level of 0.005 or 0.001 has been advised for classical hypothesis tests to produce robust and reproducible results.^33^ Fewer type 1 errors, however, come at the expense of more type 2 errors, that is, failing to demonstrate an existing association or effect. This often seems to be a sensible price to pay, as false positive findings presumably may have more detrimental consequences than false negative ones. Furthermore, increasing the sample size may decrease the risk of unnecessary type 2 errors.
- If using an NHST on a weakly supported hypothesis using conventional significance levels, this should be acknowledged as a methodological study weakness. Positive findings from such an analyses should be viewed as tentative until the results are replicated and/or the hypothesis gains more empirical evidence supporting it as being likely to be true.

## 4 SUMMARY

All hypotheses are not created equal, and this is not formally considered by NHST method. Using the NHST procedure on hypotheses with poor theoretical and empirical support increases the risk of producing false positive findings that is evidence of the untrue. This has to be acknowledged when choosing methods for statistical inference as well as in the interpretation and reporting of the findings.

## FINANCIAL SUPPORT

None

## COMPETING INTERESTS

The author declare having no competing interests.
